# The Great Five—an artificial bacterial consortium with antagonistic activity towards *Pectobacterium* spp. and *Dickeya* spp.: formulation, shelf life, and the ability to prevent soft rot of potato in storage

**DOI:** 10.1007/s00253-020-10550-x

**Published:** 2020-03-26

**Authors:** Tomasz Maciag, Dorota M. Krzyzanowska, Sylwia Jafra, Joanna Siwinska, Robert Czajkowski

**Affiliations:** 1 Division of Biological Plant Protection, Intercollegiate Faculty of Biotechnology, University of Gdansk and Medical University of Gdansk, Gdansk, Poland; 2 Division of Plant Protection and Biotechnology, Intercollegiate Faculty of Biotechnology, University of Gdansk and Medical University of Gdansk, Gdansk, Poland; 3 Division of Biologically Active Compounds, Intercollegiate Faculty of Biotechnology, University of Gdansk and Medical University of Gdansk, Gdansk, Poland

**Keywords:** Pectinolytic *Erwinia*, Blackleg, Biological control, Antagonism, Potato

## Abstract

**Abstract:**

“The Great Five” (GF) is an artificial bacterial consortium developed to protect potato tubers from soft rot caused by *Pectobacterium* spp. and *Dickeya* spp. To investigate the commercialization potential of the GF, we developed liquid and powder formulations of the consortium and of each of the comprising strains (*Serratia plymuthica* strain A294, *Enterobacter amnigenus* strain A167, *Rahnella aquatilis* strain H145, *Serratia rubidaea* strain H440, and *S. rubidaea* strain H469). To form powders, the cells were lyophilized using a newly developed lyoprotectant: Reagent PS. The shelf life of the formulations stored at 8 and 22 °C was monitored for a period of 12 months. The longest shelf life was obtained for formulations stored at 8 °C; however, the viability of all formulations was negatively affected at 22 °C. For the consortium, a 2.5 log_10_ cfu (colony forming units) drop in cell number was recorded for the liquid formulation after 6 months, while in case of powders, the drop remained below 1 log_10_ cfu following 12 months. The ability of the powder formulations to preserve biocontrol activity of the consortium was tested on potato tubers treated with the formulations and a mixture of the soft rot pathogens. The inoculated tubers were stored for 6 months at 8 °C to mimic commercial storage conditions. Soft rot severity and incidence on potato tubers treated with formulations were significantly reduced (62–75% and 48–61%, respectively) in comparison to positive control with pathogens alone. The potential use of the newly developed formulations of “The Great Five” for the biocontrol of soft rot is discussed.

****Key Points**:**

• *An innovative reagent to protect bacterial cells during lyophilization was developed*.

• *Powder formulations of “The Great Five” prolonged its shelf life*.

• *The powder-formulated “The Great Five” was active against soft rot bacteria on potato tubers*.

**Electronic supplementary material:**

The online version of this article (10.1007/s00253-020-10550-x) contains supplementary material, which is available to authorized users.

## Introduction

Pectinolytic Soft Rot *Pectobacteriaceae* (SRP: *Pectobacterium* spp. and *Dickeya* spp.; former pectinolytic *Erwinia* spp.) infect a number of plant species worldwide including agriculturally relevant crops (Toth et al. 2003). SRP are recognized among the top 10 most important bacterial pathogens in agriculture (Mansfield et al. 2012). In potato, these pathogens cause a variety of disease symptoms including pre-emergence decay of tubers, aerial stem rot, and blackleg under field conditions, as well as soft rot of progeny tubers in storage (Pérombelon 2002). In Europe, the high losses in (seed) potato production are predominantly associated with declassification and rejection of lots. This includes reduction of the market value due to the infestation with SRP (Toth et al. 2011).

It is widely accepted that the major source of SRP in the environment are latently infected potato tubers (Pérombelon 1974). Latently infected tubers can carry a relatively high SRP inoculum reaching after storage period even 10^2^–10^4^ viable cell per gram of tuber tissue. This inoculum is enough to cause soft rot symptoms in the next growing season (Czajkowski et al. 2009). Latent infections promote transmission of the inoculum as the pathogens may spread unnoticed through several generations of tubers before the occurrence of disease symptoms (Pérombelon 1992). Consequently, the production of pathogen-free seed material and/or application of protective measures against contamination with *Pectobacterium* spp. and *Dickeya* spp. remain of utmost importance (Pérombelon 1992).

A well-recognized method to reduce tuber contamination with SRP is the use of pathogen-free seed material derived from axenic potato cultures (Gopal et al. 1998). However, the use of SRP-free planting material at startup does not prevent infections that may occur later during potato cultivation in the field (Pérombelon 1974). Apart from the infected potato plants in the field and/or soft rotting tubers in storage that spread inoculum to healthy tubers, the potential reservoirs of the pectinolytic bacteria include common weeds (Tsror et al. 2010) and non-host plants (Fikowicz-Krosko et al. 2017; Toth et al. 2011), surface water (Cappaert and Powelson 1987; Harrison et al. 1987; McCarter-Zorner et al. 1984), and soil (Toth et al. 2003). Because the pathogen inoculum required to establish an infection in potato is low (Toth et al. 2003), the initially SRP-free seed tubers can rapidly become symptomatic following planting in an “open environment” (Charkowski 2015; Toth et al. 2011).

Aside from hygiene measures to prevent contamination of plant material with SRP (Czajkowski et al. 2011), potato tuber treatments may be recognized as an additive approach to increase the quality of the potato (seed) lots. Physical and/or chemical tuber treatments developed for pathogen control measures were reported to provide some level of control of infections caused by *Pectobacterium* spp. and *Dickeya* spp. in potato (Mills et al. 2006; Ranganna et al. 1997). Many of these treatments, however, show considerable phytotoxicity to the treated tubers, therefore decreasing their viability, and are difficult to apply on a large scale in the potato production systems (Pérombelon 1992), or are unable to target SRP cells localized deep in the vascular tissues (Czajkowski et al. 2011).

An environmentally friendly alternative to chemical and/or physical tuber treatments is biological control. Microbial biocontrol agents reduce the population size of the pathogens and/or suppress their virulence using different antagonistic mechanisms (Compant et al. 2005). Several publications describe the isolation and characterization of bacterial antagonists effectively controlling potato tubers against *Pectobacterium* spp. and *Dickeya* spp. (Czajkowski et al. 2012; Des Essarts et al. 2016; Jafra et al. 2009; Krzyzanowska et al. 2019b, 2012b). However, none of the biocontrol agents reported so far have been used as commercial products against SRP (Czajkowski et al. 2011).

One of the major challenges in commercial application of microbial biocontrol agents is the formulation of the working microbial inoculum (Stephens and Rask 2000). Successful formulations must enable biological control agents to remain viable during long-term storage and then become metabolically active in the environment upon application (Bashan et al. 2014). Choosing an efficient method to formulate a biocontrol strain is not trivial. Due to the variety of microbial species with different biocontrol activities, no universally applicable method exists to preserve the viability of bacterial strains used in biocontrol products (Stephens and Rask 2000). Furthermore, the final product must be compatible with the requirements of the target cropping system, safe, and relatively inexpensive to use (Bashan et al. 2014; Fravel 2005).

Microbial formulations can be prepared as solids or liquids (Berninger et al. 2018). Solid-state formulations include powders and granules, whereas liquid formulations comprise microbial cells suspended in water-based or oil-based carriers (Stephens and Rask 2000). Each of the formulation types offers different benefits. For example, granules containing encapsulated bacteria enable stable release of the microorganisms to the environment and are therefore advantageous for soil applications where timed release of the antagonists is critical (Bashan 1986). Powder formulations containing desiccated bacteria usually offer the best shelf life of the formulated inoculum (Meng et al. 2008). Finally, liquid formulations, although not as stable as solids and, in general, offering shorter shelf life of the inoculum, are the most straightforward to prepare, they are readily compatible with most agricultural equipment and are fit also for foliar application (Bashan et al. 2014).

Recently, we developed and described a (artificial) consortium of five antagonistic bacterial strains, designated the Great Five (GF), comprising: *Serratia plymuthica* strain A294, *Enterobacter amnigenus* strain A167, *Rahnella aquatilis* strain H145, *Serratia rubidaea* strain H440, and *S. rubidaea* strain H469. The GF consortium has been developed in our former study after several rounds of experiments in which consortia containing random selection of antagonistic bacterial strains were created and evaluated for protection of potato tubers against SRP. After each round, the best candidates were selected from the starting consortia and finally combined into the new GF consortium, which has been then extensively evaluated again against a mixture of SRP on potato tubers under disease-favorable conditions and with high pathogen load (Krzyzanowska et al. 2019a, b).

The GF consortium efficiently protects potato tubers from soft rot caused by a combination of SRP pathogens and under conditions promoting disease development (Krzyzanowska et al. 2019a, b). In experiments where inoculated tubers were treated with suspension of freshly grown bacterial cells, followed by immediate transfer to disease-favoring conditions (high temperature, high humidity), the GF consortium reduced soft rot incidence by as much as 46% in comparison with the control, which comprises tubers inoculated with a mixture of SRP pathogens.

In this study, we aimed to develop a formulation of the GF consortium that could be applied to the surface of potato tubers prior to storage and/or before planting to protect them against SRP. A set of different formulations was prepared and evaluated in terms of preserving bacterial viability (shelf life of the formulated bacterial strains) at two different temperatures, 8 and 22 °C, for a total period of 12 months. The formulations were also tested for the ability to suppress soft rot symptoms on treated tubers following 6-month storage in a cold room (8 °C). Moreover, as the preparation of the solid powder formulations required desiccation of cells with minimal loss of viability, we developed an innovative lyoprotectant and evaluated its efficacy in preserving cells during freeze drying. The results of the study and their implications for the biocontrol of SRP in potato with artificial (and formulated) microbial consortia are discussed.

## Materials and methods

### Bacterial strains and culture conditions

Bacterial strains used in this study are listed in Table 1. The SRP pathogens and the biological control strains of the Great Five consortium (GF): *S. plymuthica* strain A294 (Polish Collection of Microorganisms, Wroclaw, Poland (PCM) B/00143), *E. amnigenus* strain A167 (PCM B/00145), *R. aquatilis* strain H145 (PCM B/00144), *S. rubidaea* strain H440 (PCM B/00141), and *S. rubidaea* strain H469 (PCM B/00142) were grown for 24–48 h at 28 °C on Tryptone Soy Agar (TSA; Oxoid, Basingstoke, UK) or in TSB. Liquid bacterial cultures were agitated (150 rpm) during cultivation. The probiotic microorganisms (*Bacillus coagulans* (Colinox), *Lactobacillus brevis* 269Y, *Lactobacillus rhamnosus* GG, *L. rhamnosus* 573, and yeast *Saccharomyces boulardi* (ENTEROL 250)), were grown at 37 °C in De Man, Rogosa, and Sharpe medium (MRS, BTL Ltd., Warsaw, Poland), and *Eschericha coli* strain DH5α, grown in Tryptone Soy Broth (TSB; Oxoid, Basingstoke, UK) for 24–48 h at 37 °C.

**Table 1:** Bacterial strains used in this study

Species	Strain	International culture collection no.	Reference/source
Strains of the GF consortium of antagonists			
*Enterobacter amnigenus*	A167	PCM B/00145	(Jafra et al. 2006)
*Rahnella aquatilis*	H145	PCM B/00144	(Jafra et al. 2009)
*Serratia plymuthica*	A294	PCM B/00143	(Jafra et al. 2006)
*Serratia rubidaea*	H440	PCM B/00141	(Jafra et al. 2009)
*Serratia rubidaea*	H469	PCM B/00142	(Jafra et al. 2009)
SRP bacteria comprising the mix of five plant pathogens			
*Pectobacterium atrosepticum*	SCRI 1043	ATCC BAA-672	(Bell et al. 2004)
*Pectobacterium carotovorum* subsp. *carotovorum*	Ecc71	ATCC 15713	(Willis et al. 1987)
*Pectobacterium parmentieri*	SCC3193	CFBP 8475, LMG 29774, LMG:29774	(Pirhonen et al. 1991)
*Dickeya solani*	IPO2222	DSM 28711, LMG 25993, NCPPB 4479	(van der Wolf et al. 2014)
*Dickeya dianthicola*	CFBP 1200	NCPPB 453 T, ICMP 6427 T LMG 2485 T	(Samson et al. 2005)
Probiotic microorganisms supplemented to humans			
*Bacillus coagulans*	Not indicated	Not indicated	Colinox (VITAMED)
*Lactobacillus brevis*	269Y	DSM 20556, ATCC 8287	(Davis 1955)
*Lactobacillus rhamnosus*	GG	ATCC 53103	(Goldin et al. 1992)
*Lactobacillus rhamnosus*	573	Not indicated	Lactovaginal (IBSS Biomed)
*Saccharomyces boulardi* (yeast)	not indicated	Not indicated	ENTEROL 250 (BIOCODEX)
Plant-associated bacteria			
*Ochrobactrum quorumnocens*	A44	LMG 30544 PCM 2957	(Krzyzanowska et al. 2019a, b)
*Pseudomonas donghuensis*	P482	CCTCC AB 2012141 NRRL B-59108	(Krzyzanowska et al. 2012a)
*Pseudomonas protegens*	CHA0	DSM 19095 LMG 27888	(Stutz et al. 1986)
Other			
*Bacillus subtilis*	168	BGSC 1A700	(Ehrenberg 1834)
*Escherichia coli*	DH5α	NCTC 13450	(Anthony and Bailey 1989)

*GF* the Great Five; a consortium of bacterial antagonists (A294, A167, H145, H440, H469) shown to attenuate soft rot caused by the SRP pathogens (Krzyzanowska et al. 2019a, b*PCM*, Polish Collection of Microorganisms; Wroclaw, Poland, https://www.pcm.org.pl/home, patent deposit according to the Budapest treaty; *SRP*, Soft Rot *Pectobacteriaceae*, plant pathogenic bacteria of genera *Dickeya* and *Pectobacterium*, causing soft rot and blackleg diseases on vegetables and ornamental plants

### Freeze drying (lyophilization) of bacterial cells

Small-scale lyophilization (up to 80 mg fresh weight) was performed to evaluate how the studied biological control strains comprising the GF consortium of antagonists (A294, A167, H145, H440, and H469) survive the freeze-drying procedure in the presence and absence of lyoprotectants. To provide a point of reference, the experiment was additionally carried out on 10 other microorganisms: three plant-associated bacterial strains (*Ochrobactrum quorumnocens* A44 (Krzyzanowska et al. 2019a, b), *Pseudomonas donghuensis* P482 (Krzyzanowska et al. 2012a), and *Pseudomonas protegens* CHA0 (Stutz et al. 1986), five probiotic microorganisms (*B. coagulans*, *L. brevis* 269Y, *L. rhamnosus* GG, *L. rhamnosus* 573, and yeast *S. boulardi*), and two other well-studied model bacterial strains: *Bacillus subtilis* 168 and *E. coli* DH5α (Tab.1). The two tested lyoprotectants included Reagent 18, recommended for freeze drying of microorganisms by the American Type Culture Collection (ATCC) (per 100 mL: 0.75 g TSB, 10 g sucrose, 5 g bovine serum albumin (BSA)), and Reagent PS—a modified version of Reagent 18 in which a bovine serum albumin (BSA) was replaced with a wheat peptone (per 100 mL: 0.75 g TSB, 10 g sucrose, 0.255 g wheat peptone (Sigma-Aldrich, Darmstadt, Germany) (Polish patent application P.428215, 2018).

For small-scale lyophilization, cells from 1 mL of overnight bacterial cultures were pelleted (4500×g, 5 min) and weighted. Each 250 mg of cell fresh weight (fw) was re-suspended in 1 mL of either Reagent 18, Reagent PS, or sterile distilled water (negative control). Cell suspensions representing all 45 combinations (15 strains, each in 3 lyophilization media) and in three technical replicates each (*n* = 135) were frozen overnight at − 80 °C and subsequently freeze dried for 24 h at − 50 °C in the Heto PowerDry Freeze Dryer (Thermo Scientific, Warsaw, Poland). The dry pellets were thoroughly re-suspended in sterile distilled water, in a volume equal to the initial volume of the sample (1 mL). The number of viable cells was determined before and after freeze drying by plating 10 μl 10-fold serial dilutions of bacterial suspensions on a suitable growth medium—MRS agar for probiotic microorganisms and TSA for all other strains. Each dilution was plated in three technical replicates. Per strain and lyophilization medium, cell survival rate was calculated according to the equation:

$$ \mathrm{survival}\ \mathrm{rate}\ \left(\%\right)=\frac{\mathrm{cfu}\ {\mathrm{g}}^{-1}\mathrm{fw}\ \mathrm{after}\ \mathrm{freeze}\ \mathrm{drying}}{\mathrm{cfu}\ {\mathrm{g}}^{-1}\ \mathrm{fw}\ \mathrm{before}\ \mathrm{freeze}\ \mathrm{drying}}\times 100\% $$

where cfu—colony forming units; fw—cell fresh weight (Miyamoto-Shinohara et al. 2006).

Large-scale lyophilization (up to 80 g fresh weight) of the GF strains A294, A167, H145, H440, and H469 was outsourced to Pomeranian Science and Technology Park in Gdynia (PPNT, Gdynia, Poland). As a part of this service, the bacterial isolates were individually cultured in 10 L of TSB and freeze dried in 100 mL of Reagent PS per 25 g of bacterial fresh weight (fw). For each strain, the procedure was performed twice, yielding two independent batches of freeze-dried cells. The lyophilizates were stored in glass jars in the presence of silica gel desiccant, at 8 °C in the dark.

### Formulation

Each strain of the GF consortium (A294, A167, H145, H440, and H469) was formulated into two wettable powders (WPs), one liquid preparation (LQ), and a control (CTRL). The WPs comprised of bacterial lyophilizate, a carrier: kaolinite (ZielonyKlub.pl, Poland) or diatomaceous earth (Perma-Guard, Otwock, Poland), and a common mix of chemicals reported in literature to increase shelf life of bacterial formulations (Arora and Mishra 2016), that is: methyl cellulose 15 cP (mPa s) (Sigma-Aldrich, Darmstadt, Germany), cyclodextrin (Sigma-Aldrich, Darmstadt, Germany), sodium lignosulphonate (Roth, Karlsruhe, Germany, and KH_2_PO_4_ (POCH, Warsaw, Poland). The wettable powder (WP) with the kaolinite carrier was designated WP-KAO, and the WP with diatomaceous earth carrier was designated WP-DE. The composition (in %) of all formulations is given in Table 2.

**Table 2:** Composition of the tested bacterial formulations

Component^a^	Liquid		Powder (dry)		
	CTRL	LQ	LYO	WP-KAO	WP-DE
Mix of GF strains^b^	100%	89.9%	100%	60%	60%
	(suspension)	(suspension)	(lyophilizate)	(lyophilizate)	(lyophilizate)
Solid carrier^c^	–	–	–	29.9%	29.9%
Methyl cellulose 15 cP	–	5%	–	5%	5%
Cyclodextrin	–	0.1%	–	0.1%	0.1%
Sodium lignosulphorate	–	4%	–	4%	4%
KH_2_PO_4_	–	1%	–	1%	1%

^a^The percentages are given in or w/v ratios for LQ and in w/w ratios for WP-KAO and WP-DE

^b^Bacterial strains were mixed in equal v/v or w/w ratios. In all combinations, the total titer of bacterial cells in the final formulation/control equaled ca. 5 × 10^10^ cfu mL^−1^ in the liquid suspensions (CTRL, LQ) and 1 × 10^11^ cfu g^−1^ in powders (LYO, WP-KAO, WP-DE). Cell suspensions were prepared in 1/4 Ringer’s buffer and the lyophilizates were obtained using the PS lyoprotectant

^c^In case of WP-KAO, the solid carrier was kaolinite. In case of WP-DE, the carrier was diatomaceous earth; “–” component not added to a given consortium

To prepare the liquid formulation, designated LQ cells were freshly cultured overnight on TSA at 28 °C, harvested by scraping them from the agar, and suspended in 1/4 Ringer’s buffer (Merck, Darmstadt, Germany) to obtain the turbidity of 15 McF (ca. 5 × 10^10^ cfu mL^−1^). The suspension was supplemented with 100 mg mL^−1^ of LQ formulation mix (49.5% methyl cellulose 15 cP, 0.99% cyclodextrin, 39.6% sodium lignosulphonate, 9.9% KH_2_PO_4_). The 100 mg of this formulation mix was added per 1 mL of bacterial suspension which resulted in a final concentration of the additives analogous to that applied for the WPs (Table 2). Fresh bacterial suspensions in 1/4 Ringer’s buffer but without the formulation mix were used as control (CTRL).

To reliably determine the shelf life of the formulations, defined as the viability of bacteria over time (Berninger et al. 2018), two individual lots were prepared for each formulation, with a postponement between them of 8 months. For powder formulations, each lot comprised of cells freeze-dried in independent procedures.

### Viability of the antagonistic strains of GF during long-term storage

The shelf life of individual strains: A294, A167, H145, H440, H469, and their mixture, the GF consortium was investigated in two liquid and three powder preparations. The liquid preparations included the LQ formulation and the control suspensions in 1/4 Ringer’s buffer (CTRL). The powder preparations included the lyophilizate (LYO) without the addition of formulation mix and two lyophilizate-based WPs: WP-KAO and WP-DE.

To store powder formulations for the shelf life experiment, the formulations (LYO, WP-KAO, or WP-DE) were placed in a sterile 5-mL Eppendorf tube. The samples, each in two technical replicates, placed in a plastic box with silica gel desiccant were stored at either 8 or 22 °C for a total period of 12 months.

To store liquid preparations (LQ and CTRL), 5 mL of each sample, two technical replicates each, were aliquoted into sterile wide-mouth amber glass bottles with stoppers (Bionovo, Legnica, Poland). The bottles were kept at either 8 or 22 °C for 12 months.

All formulations (LYO, WP-KAO, WP-DE, LQ) and the control (CTRL) were sampled immediately after preparation and, subsequently, following 1, 2, 3, 6, 9, and 12 months of storage at both temperatures (8 and 22 °C). At the respective time points, aliquots of the powder formulations (LYO, WP-KAO, and WP-DE) (ca. 100 mg) were collected with a sterile spatula, weighed, and thoroughly re-suspended in 1 mL of sterile distilled water per each 100 mg of the powder. Aliquots of 100 μL were collected in case of the liquid preparations (LQ and CTRL). The viability of the cells in each preparation was determined by dilution plating on TSA as described above for the assessment of the efficiency of lyoprotectants. Results were expressed in cfu g^−1^ for the dry and in cfu mL^−1^ for the liquid preparations. In order to compare the shelf life of different formulations, slope of the survival curve (*x*), expressing the average decline in log_10_ cfu per month of storage, was calculated with the following equation:

$$ x=\frac{\sum \left({t}_i-\overline{t}\right)\left({y}_i-\overline{y}\right)}{\sum {\left({t}_i-\overline{t}\right)}^2} $$

where *y*_*i*_ stands for the count of viable cells (log_10_ cfu g^−1^ or log_10_ cfu mL^−1^) at the corresponding time of sampling (*t*_*i*_, in months), $\overline{y}$ is the average of all *y*_*i*_ values, and $\overline{t}$ is the average of all *t*_*i*_ values. The lower the slope value, the steeper decrease in the number of viable cells was observed over time. For storage at 8 °C, the whole experiment was performed twice, separately for each lot of formulations (lot 1 and lot 2). For 22 °C, due to low cell survival in the first experiment, the experiment was not repeated.

### Biocontrol efficacy of bacterial preparations—protection of potato tubers against SRPs in storage

High-quality, pathogen-free seed tubers cv. Irga (caliber 30–50 mm), showing moderate resistance to soft rot (4.0 in 9.0 rank scale; http://ziemniak-bonin.pl), were purchased from a potato breeding company (Pomorsko-Mazurska Hodowla Ziemniaka, ang. Pomeranian-Masurian Potato Breeding Szyldak, Poland, http://www.pmhz.pl/). The potato tubers cv. Irga expressing moderate resistance to soft rot were chosen to mimic the natural field/storage situation. The moderate resistant cultivars are commonly used by farmers worldwide. The susceptible cultivars are not used commercially due to the high soft rot incidence and consequently high losses. Soft rot immune (fully resistant) potato cultivars do not exist on the market (Czajkowski et al. 2011).

The tubers were subsequently treated, by vacuum infiltration (Czajkowski et al. 2012) with the GF consortium of antagonists (A294, A167, H145, H440, and H469 in equal ratios) and a composition of five soft rot pathogens of genera *Pectobacterium* and *Dickeya* (*Pectobacterium atrosepticum* strain SCRI 1043, *Pectobacterium carotovorum* subsp. *carotovorum* strain Ecc71, *Pectobacterium parmentieri* strain 16 SCC3193, *Dickeya solani* strain IPO2222, and *Dickeya dianthicola* strain CFBP 1200 (IPO1741), representing species and subspecies known to most often cause soft rot and blackleg diseases in Europe (Pérombelon 2002; van der Wolf et al. 2017). Prior to the other experiments, the virulence of *Pectobacterium* spp. and *Dickeya* spp. strains used in this study was verified using potato slice assays as described in (Czajkowski et al. 2012).

The bacterization of tubers was performed according to a protocol modified from Krzyzanowska et al. (2019a, b). The modifications included an additional storage period in the cold room (8 °C) and the scale of the experiment (number of tubers processed per combination). Briefly, potato tubers were surface sterilized for 20 min in 5% commercial bleach (ACE, Procter and Gamble, Gdansk, Poland), washed 3 times with running tap water, and placed in 5-L beakers, 30 tubers each. Next, the tubers were immersed in the suspensions of the antagonists formulated as described above. The tested preparations included the lyophilizate (LYO), two WPs (WP-KAO and WP-DE), and unpreserved cells from fresh cultures on TSA medium suspended in ¼ Ringer’s buffer (FR). The metabolically active cells as present in FR were not previously tested in a long-term storage experiment. Moreover, FR provides a point of reference for the performance of formulated (preserved) cells in LYO, WP-KAO, and WP-DE. Working solutions of dry preparations were obtained by adding 500 mg of powder to 1 L of tap water (ca. 5 × 10^8^ cfu mL^−1^ of antagonistic bacteria in total, based on the count of viable cells in the stored formulations at the time of the experiment). The FR suspension (also 5 × 10^8^ cfu mL^−1^ of bacteria in total), as well as the mix of five soft rot pathogens (1 × 10^6^ cfu mL^−1^ of bacteria in total, 5 × 10^5^ cfu mL^−1^ of each individual strain), were prepared as described in Krzyzanowska et al. (2019a, b). The immersed tubers were placed in a desiccator and vacuum-infiltrated at – 80 Bar for 10 min, followed by incubation for an additional 10 min under atmospheric pressure to facilitate the penetration of bacteria into lenticels and wounds of the tubers. Inoculated potato tubers were air-dried overnight and, on the following day, vacuum-infiltrated with the mixture of five soft rot pathogens. Following the second round of vacuum infiltration, potato tubers were air-dried for 1.5 h and placed in covered, nontransparent plastic boxes, 30 tubers per box (box dimensions 26 × 18 × 12 cm). For the control treatments, tubers were inoculated with tap water and the pathogens (positive control for the occurrence of soft rot, PC) or tap water alone (negative control, NC). Each preparation was tested in five technical replicates (*n* = 5 boxes × 30 tubers = 150 tubers). Boxes were stored for 6 months in a cold room, at 8 °C, 80% relative humidity, to simulate conditions applied for the storage of potato tubers (2–10 °C, depending on storage time and tuber type) (Beukema and van der Zaag 1990; Bradshaw and Ramsay 2009). Following the period of storage, the emerging sprouts, if present, were removed from the tubers, and the sprout-less tubers were incubated for 5 days under disease-favoring conditions (28 °C, 85–90% relative humidity), 15 tubers of the same treatment per box, to initiate soft rot. The temperature of 28 °C and 90% relative humidity were chosen to stimulate expression of soft rot symptoms caused by SRP on potato tubers. This experimental setup assured the worst case scenario, in which potato tubers of the relatively susceptible potato cultivar were challenged with high inoculum of the mixture of SRP pathogens under conditions stimulating the development of infection symptoms. The formulations containing the GF consortium able to protect tubers under disease-favorable conditions should confer their efficacy also under conditions less suitable for disease development (e.g., commercial storage conditions).

Severity of soft rot symptoms was assessed individually for each tuber using a six-rank disease severity scale (0–5) as previously described (Krzyzanowska et al. 2019a, b: 0—no symptoms, 1—rotting symptoms localized in the periderm and overall on less than 25% of tuber surface, 2—symptoms as in rank 1 but present on 25 to 50% of tuber surface, 3—symptoms as in rank 2 but with the periderm detaching from the internal tissue of the tuber (core) and the rotting occupying between 50 and 90% of the tuber, 4—symptoms as in rank 3 but overall the rotting occupies more than 90% of the surface and spreads to the core, and 5—maceration of the whole tuber. The experiment was performed twice. In total, each treatment was evaluated on 300 seed tubers, yielding a total of 1800 tubers for 6 treatments tested.

### Statistical analyses

Statistical analyses were performed with R version 3.5.1 (RTeam 2018) using the RStudio (2017). For the evaluation the survival of cells following freeze drying, as well as for the shelf life experiment, the normality of distribution of the residuals was tested with Shapiro-Wilk test, and the homogeneity of variance was tested with Levene’s test included in the “car” package (Fox and Weisberg 2010). For data with normal distribution and non-homogenic variance, as observed in case of the shelf life experiment, the differences between multiple groups were analyzed with Welch’s unequal variances *t* test, followed by pairwise comparisons using Games-Howell test from “PCMRplus” package (Bürkner 2017). For data with non-normal distribution, as obtained in the experiment concerning the survival of cells following freeze drying, Kruskal-Wallis test (Kruskal and Wallis 1952) was used to determine the differences between samples, followed by a post hoc Dunn’s test from the “dunn.test” package (Dinno 2017). The latter approach was also applied for the analysis of data in ordinal scale, like the ranks in disease severity scale (0–5) obtained in the biocontrol experiment on potato tubers. For data on the incidence of soft rot, expressed in binominal scale (0—lack of symptoms, 1—occurrence of symptoms), the Chi^2^ test was applied to determine the difference between the expected and the observed frequencies between sample sets. For data from shelf life experiment, regarding survival at different temperatures, the normality of distribution of the data was checked with Shapiro-Wilk test. Due to non-normal distribution of data, Wilcoxon signed rank test was used to investigate differences between groups (Wilcoxon 1947).

## Results

### The GF strains show high survival rate following freeze drying in Reagent PS—a BSA-free alternative to the Reagent 18

The count of viable cells before and after the freeze drying procedure and the ratio was calculated to assess how the biocontrol strains of the GF consortium (*S. plymuthica* strain A294, *E. amnigenus* strain A167, *R. aquatilis* strain H145, *S. rubidaea* strain H440, and *S. rubidaea* strain H469) survive the freeze drying procedure and if they can be successfully preserved for long-term storage using this method. To provide a reference point, the ten other microorganisms were tested in parallel (4 probiotic bacteria, 1 probiotic yeast, 3 plant-associated bacteria, and the model *B. subtilis* strain 168 and *E. coli* strain DH5α).

**Fig. 1:**
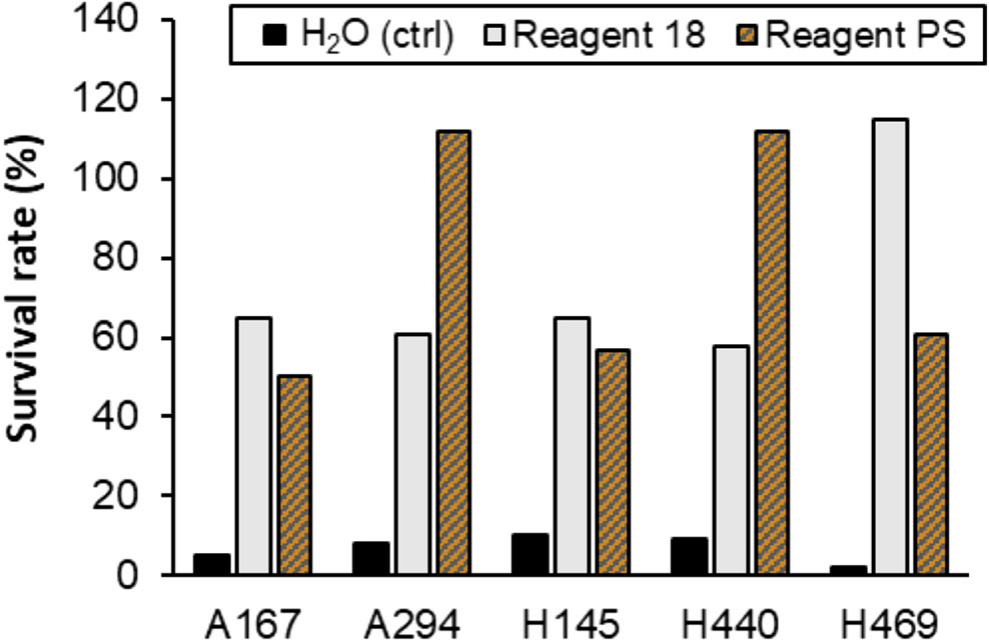
Average survival rate of the tested GF strains following freeze drying in water (control) and in two lyoprotectants: Reagent 18 and Reagent PS. CTRL, control

The count of viable cells in the starting suspensions (prior to freeze drying) was approx. 10–11 log_10_ cfu g^−1^ (10^10^ to 10^11^ cfu g^−^1) of cell fresh weight (fw) for all 14 bacterial strains and approx. 9 log_10_ cfu g^−1^ fw (1 × 10^9^ cfu g^−1^) for the single yeast strain tested. Following lyophilization, the positive effect of applying lyoprotectants was visible for 13 out of 15 tested microorganisms, providing cell survival between 6 and ca. 100 times higher in comparison to the survival of cells freeze-dried in water (negative control) (Supplementary Table S1). For the five biocontrol strains of the GF, the survival rate in Reagent 18 was ≥ 58% (average 73%) and in Reagent PS ≥ 57% (average 78%), and only 2–10% (average 7%) in water (Fig. 1), equaling a 1–1.7 drop in log_10_ cfu g^−1^ fw (Supplementary Table S2). The highest survival rate among the GF strains was obtained for *S. plymuthica* A294 and *S. rubidea* H440 in Reagent PS and for *S. rubidea* H469 in Reagent 18. In these cases, no loss of viable bacterial cells was detected (Fig. 1, Supplementary Table S1). At the same time, even with the addition of lyoprotectants, the survival rate of *P. protegens* CHA0, widely studied for its biocontrol properties, never exceeded 60%.

The most dramatic increase of survival upon the application of lyoprotectants was observed for the 3 tested probiotic *Lactobacilli*: two *L. rhamnosus* strains (GG and 573) and *L. brevis* 269Y. In case of these strains, the survival rate was improved 14–60-fold from 1 to 5% in water to 60–72% in Reagent 18; in comparison, almost no loss of viability was observed in Reagent PS. On the contrary, for *O. quorumnocens* A44, the increase in viability was less pronounced due to the high survival rate of the strain in water (37%). None of the tested conditions were suitable to efficiently preserve the yeast *S. boulardi* (ENTEROL 250 (BIOCODEX, Warsaw, Poland)).

**Fig. 2:**
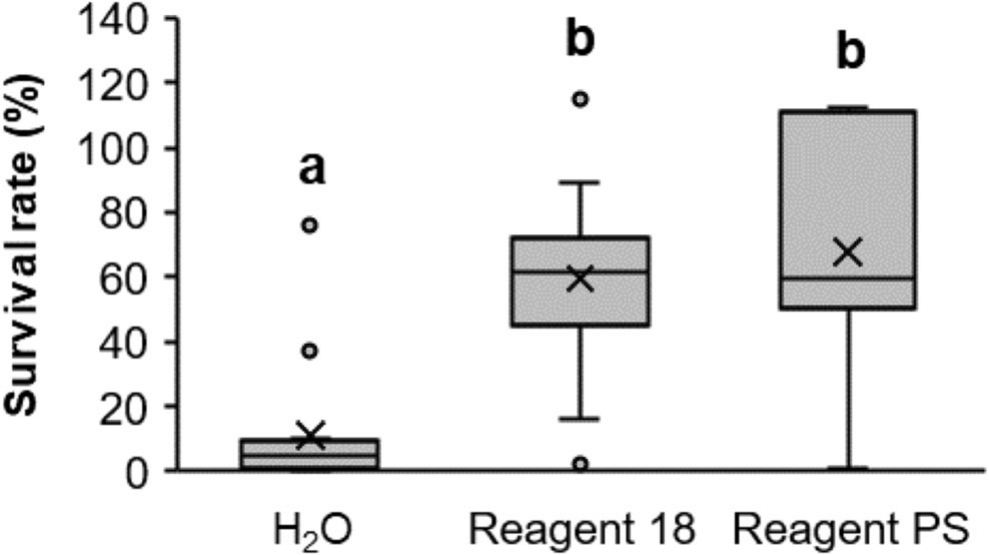
Cell survival rate following freeze drying in water (control) and in the presence of two different lyoprotectants, Reagent 18 and Reagent PS. Data for 15 microorganisms were analyzed collectively. Significantly different groups (Dunn’s test, *α* = 0.05) are marked with different letters

Along with the comparison of the survival rate of individual strains, we evaluated the overall performance of two lyoprotectants: Reagent 18 and Reagent PS developed specifically for this study. The results showed that the protective effect of Reagent PS was not significantly different (*α* = 0.05) from that of Reagent 18, with the average survival rates calculated for all 15 strains being 68% and 60%, respectively (Fig. 2). The average cell survival rate in water (11%) was significantly lower than that in Reagent 18 and Reagent PS (*p* = 0.0001 in both cases).

### The shelf life of the GF strains is higher in dry formulations stored at 8 °C than in liquid formulations stored at 22 °C

Long-term viability of the five GF strains, in different formulations, was evaluated for individual strains as well as for the five-strain consortium over a period of 12 months. The tested formulations included two liquid formulations: LQ (cells in ¼ Ringer’s supplemented with the formulation mix) and CTRL (control for LQ; cells in ¼ Ringer’s alone), and three powders: LYO (lyophilizate), WP-KAO, and WP-DE. In total, 30 combinations were tested (5 individual strains plus the GF consortium (=6) × 5 formulations). Two independent experiments were carried out involving two different formulation lots (lot 1 and lot 2). The initial count of viable cells was between 9.6 and 10.5 log_10_ cfu mL^−1^ for the liquid formulations and between 10.0 and 11.9 log_10_ cfu g^−1^ for the powder ones (Supplementary Table S3). When evaluating cell survival, the absolute change in the number of cells between the starting value and the final value, as well as the linear regression slope (trend) values of the survival curves, were considered. The slope values express the average decline in cell count (log_10_ cfu g^−1^ or cfu mL^−1^) per each month of storage. The lower the calculated slope value, the steeper decrease in the number of viable cells was observed. The comparison of the slope values, further transformed to lethality rate constants (*k*), is a good way to reliably compare the survival between different strains, formulations, and in different lots, irrespective of the initial variance in the cell count (Golowczyc et al. 2011).

**Fig. 3:**
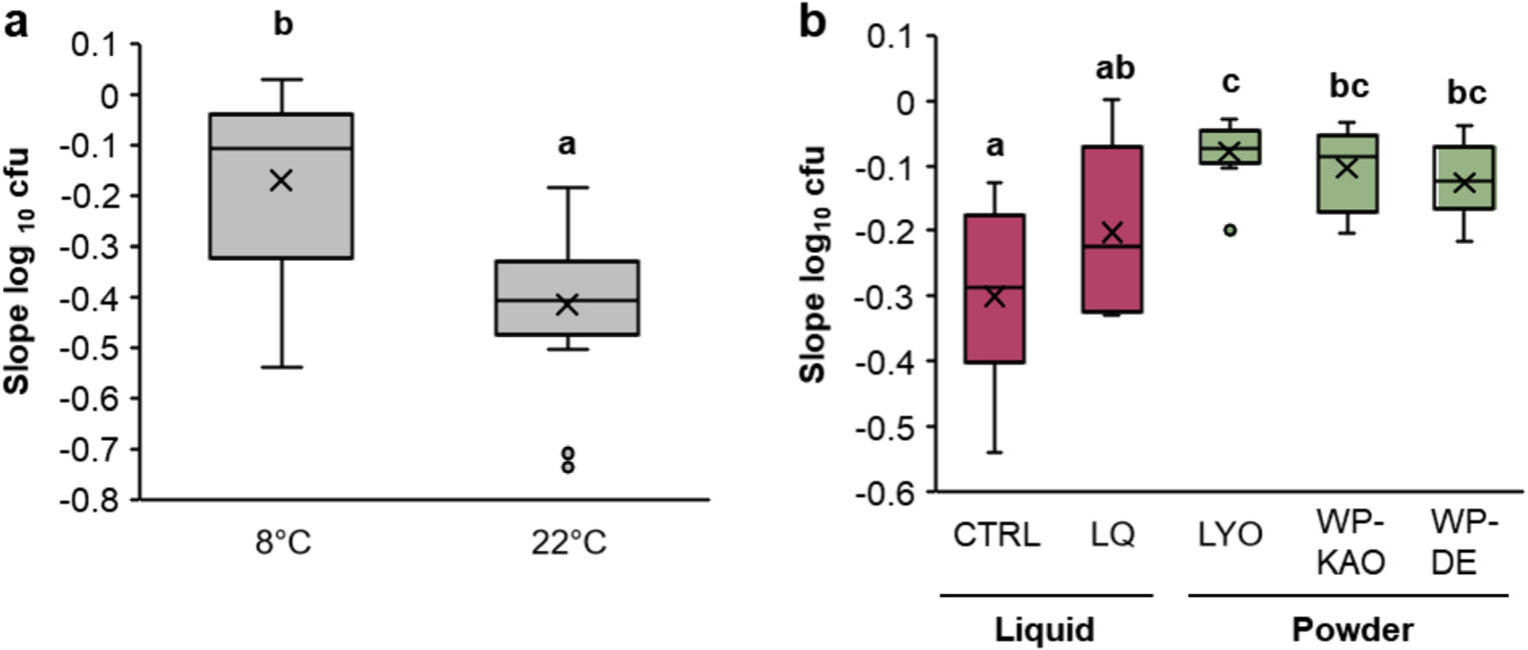
The influence of temperature (**a**) and the formulation method (**b**) on the shelf life of the GF strains. The analyses were performed on data pooled for all five GF strains. Each box shows the slope of the survival curves (change in log_10_ cfu), calculated based on the count of viable cells (cfu mL^−**1**^ for the liquid formulations and cfu g^−**1**^ for the powders) for 5 time points in panel **a** (0, 1, 2, 3, and 6 months) and 7 time points in panel **b** (additionally 9 and 12 months, with the exception of LQ and CTRL from lot 1). The higher (closer to zero) are the calculated slope values, the better is the survival rate of the strains. Liquid formulations (red boxes): CTRL, positive control—cells suspended in ¼ Ringer’s buffer (control); LQ—cells suspended in ¼ Ringer’s supplemented with the formulation mix. Powder formulations (green boxes): LYO, bacterial lyophilizates; WP-KAO—bacterial lyophilizates with WPs formulation mix, kaolinite carrier; WP-DE—bacterial lyophilizates with WPs formulation mix, diatomaceous earth. Different letters indicate significant differences between groups (*t* test, *α* = 0.05)

Bacterial viability over time was compared at two temperatures: 8 and 22 °C, mimicking cold conditions predominantly used for potato tuber storage and room temperature, respectively. Gathering of data and evaluation of the results were performed at the fifth sampling time point, that is following 6 months of storage. At 8 °C, 17 out of 30 tested combinations (57%) showed less than one order of magnitude (1 log_10_ cfu g^−1^ or cfu mL^−1^) drop in the count of viable cells, indicating a high survival rate in refrigerated conditions. At the same time point, the survival rate for all strains at 22 °C was statistically lower, with an average decline equaling nearly three orders of magnitude (2.7 log_10_ cfu g^−1^ or cfu mL^−1^). At room temperature, a decrease ≥ 1 log_10_ cfu was observed for all 30 combinations (100%), and a decrease ≥ 2 log10 cfu was observed for as many as 22 (73%) (Supplementary Table S4). In line with the above data, the slope values for the survival of strains kept at 8 °C (− 0.17; average for all strains) was significantly higher (*p* < 0.000001) than those kept at 22 °C (− 0.41) (Fig. 3a), indicating a steeper decrease in the number of viable cells at 22 °C. Considering the effect of different temperatures after 6 months of storage, the monitoring of shelf life of the tested formulations at 22 °C was discontinued due to low cell survival. The monitoring of the viability at 8 °C was continued for up to 12 months.

**Fig. 4:**
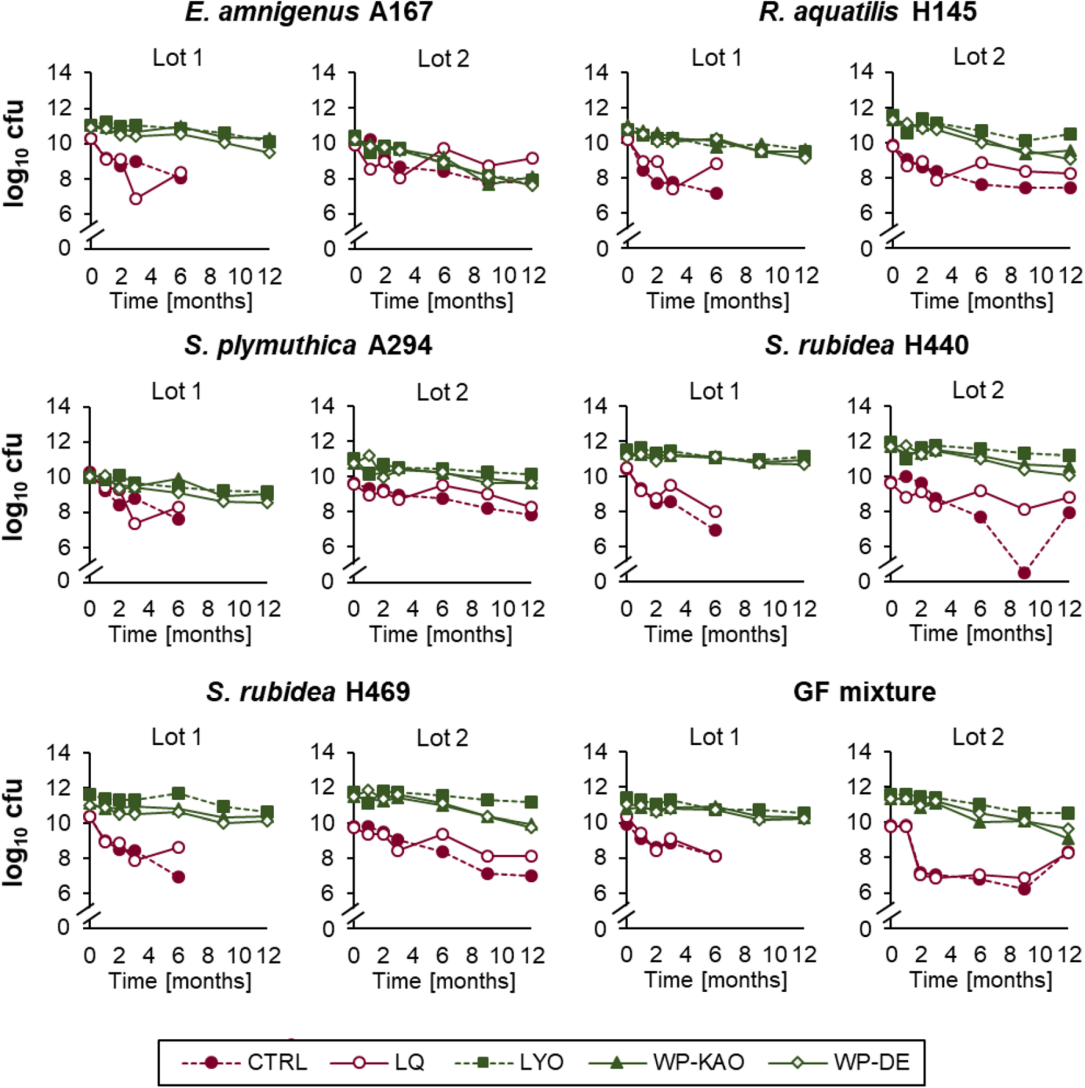
The count of viable cells of the GF antagonists in different formulations stored at 8 °C for a total period of 12 months. Lot 1 and lot 2 refer to independent experiments in which different batches of formulations were tested. CTRL (control)—cells suspended in ¼ Ringer’s buffer; LQ—cells suspended in ¼ Ringer’s supplemented with LQ formulation mix; LYO—bacterial lyophilizates; WP-KAO—bacterial lyophilizates with WPs formulation mix, kaolinite carrier; WP-DE—bacterial lyophilizates with WPs formulation mix, diatomaceous earth. The sudden drop of the *S. rubidea* H440 log_10_ cfu in lot 2 observed between 8 and 10 months in control (CTRL) is an outlier happened due to the technical error. Another cell count, proceeding the 12-month time point (not shown in the Figure) in this treatment is in line with the assessment at the 12 month

Apart from analyzing the effect of temperature, we analyzed the influence of different formulation methods on the stability of formulated consortia. The ability of different formulations to assure high survival rate of the cells stored at 8 °C was compared using data pooled for all strains formulated in a given manner. In general, the survival rate of the GF strains in dry formulations (LYO, WP-KAO, WP-DE) was very good (Fig. 4) and, based on the comparison of the slope values, significantly higher than in the liquid preparations (LQ, CTRL) (Fig. 3b). From the tested formulation methods, the CTRL (bacterial suspension in ¼ Ringer’s buffer alone) offered the lowest survival rates (− 0.28) and the lyophilizates (LYO) the highest (− 0.08) (Fig. 3b). The differences observed between the three dry formulations, LYO, WP-KAO, and WP-DE, were not statistically significant (*α* = 0.05), and the two latter (WP-KAO and WP-DE) formulations offered a considerable reduction of dusting during development of the formulations and the subsequent handling.

A strain-by-strain comparison of different formulations stored at 8 °C revealed that the most severe decline in the number of viable cells was observed for *S. rubidaea* H440 stored in ¼ Ringer’s buffer (CTRL), with the slope equaling − 0.424 and a drop of 2–3.5 log_10_ cfu mL^−1^. On the contrary, the combination offering the highest survival rate was the same strain yet preserved in a dry form of lyophilizate (LYO) (average slope = − 0.037; drop ≤ 0.4 log_10_ cfu g^−1^t following 6 months) (Supplementary Table S3).

To conclude, the most promising shelf life results were obtained for formulations containing desiccated cells stored under refrigerated conditions (8 °C).

### Powder formulations of the GF consortium significantly reduce the incidence and the severity of soft rot following 6 months of storage of inoculated potato tubers at 8 °C

To test if the formulated consortium of the GF antagonists can protect potato tubers in a setup mimicking the commercial storage conditions, tubers inoculated with both the GF and the combination of soft rot pathogens were stored for 6 months at 8 °C and subsequently transferred to disease-favorable conditions to initiate tuber rotting. A total of 2.7% of all tubers processed in Experiments 1 and 2 already showed symptoms already after their recovery from the cold room. These tubers were not transferred to disease-favorable conditions, which was the subsequent step of the experimental procedure. All tubers showing decay at 8 °C were considered as symptomatic and were assigned to the maximum severity rank 5.

**Fig. 5:**
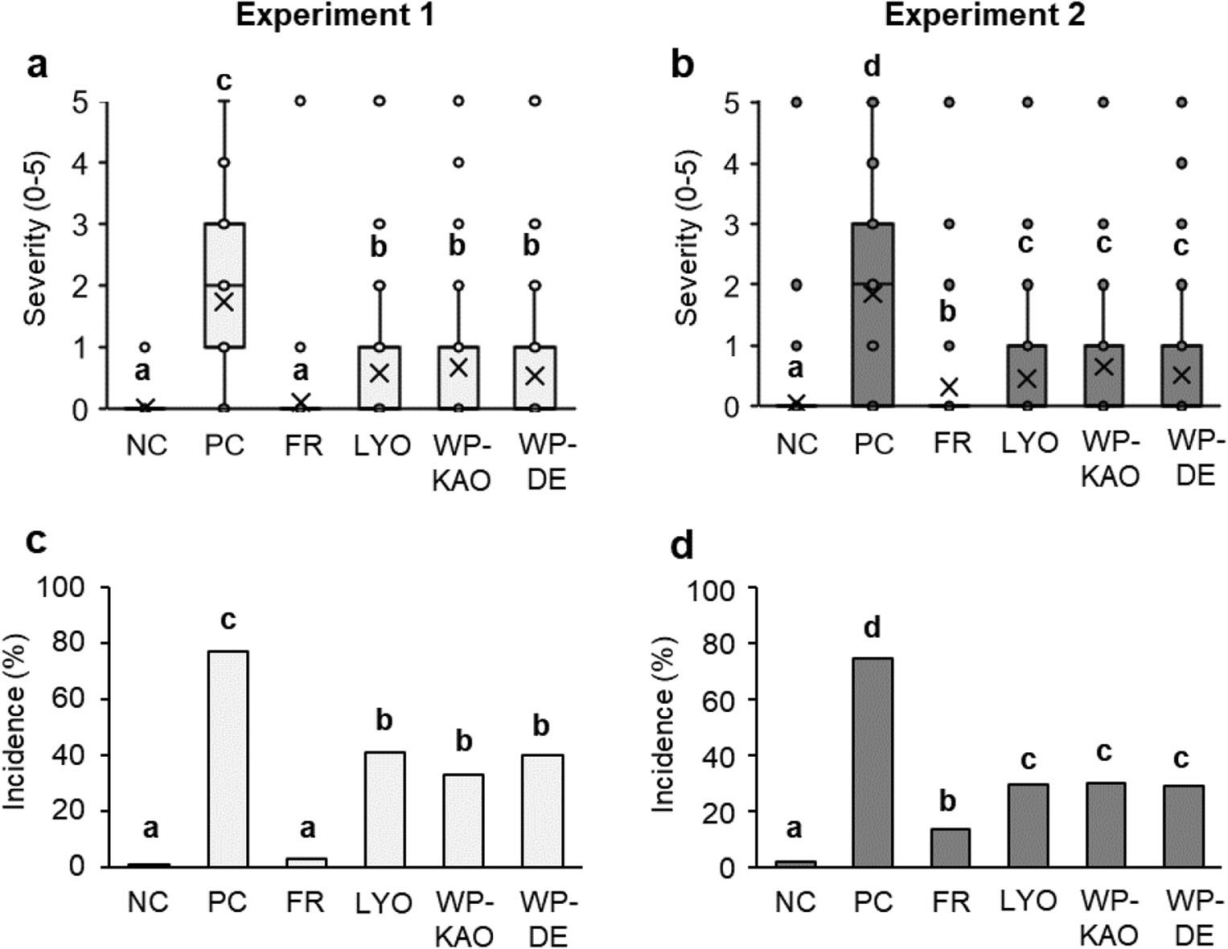
Soft rot incidence (**a**, **b**) and soft rot severity (**c**, **d**) on potato tubers infiltrated with the GF antagonists and the SRP pathogens, followed by a 6-month storage at 8 °C. Results from two independent experiments, Experiment 1 (light gray) and Experiment 2 (dark gray) are shown separately. The severity of symptoms was evaluated in a six-rank scale (0–5). NC negative control, tubers inoculated with water; PC positive control for the emergence of soft rot, tubers inoculated with SRPs alone. All other samples were co-inoculated with the GF antagonists and the SRP. Depending on the treatment, the GFs were delivered as: FR fresh cultures; LYO, lyophilizates; WP-KAO, lyophilizates formulated into a wettable powder with a kaolinite carrier; WP-DE, lyophilizates formulated into a wettable powder with a diatomaceous earth carrier. In the box plots **c** and **d**, each box determines the inter-quartile range (Q1–Q3), the line indicates the median value, the “×” stands for the average value, the whiskers indicate extreme values within 1.5 times distance from Q1–Q3, and single data points are outliners. In each experiment, a given combination was tested on 150 tubers. Different letters indicate significant differences (*α* = 0.05) between groups in Dunn’s test (a, b) or Chi^2^ (c, d)

Co-inoculation of potato tubers with the freshly grown GF antagonists (FR) reduced the average soft rot severity, in comparison to pathogen-only control (PC), by 94% in Experiment 1 and by 83% in Experiment 2. The protective efficacy of LYO, WP-DE, or WP-KAO was comparable between the three treatments and, although not as impressive as that of FR, still high, showing a 62–69% decrease in the average severity score in Experiment and 64–75% in Experiment 2 (Fig. 5a, b).

During the evaluation of disease incidence, only the tubers showing absolutely no disease symptoms were considered as healthy, whereas all other tubers were treated as symptomatic. In the pathogen-only control (PC), the soft rot incidence equaled 77% in Experiment 1 and 74% in Experiment 2. In comparison to PC, the application of FR reduced the disease incidence by 96% in Experiment 1 and by 82% in Experiment 2. Treatment with lyophilizate (LYO) reduced the incidence by 47% and 60%, and the two tested formulations, WP-KAO and WP-DE, resulted in a reduction of 48% and 59%, and 57% and 61% in comparison with the control, respectively (Fig. 5c, d.

## Discussion

Although a number of attempts have been made to control SRP on potato using biological control agents, so far, none of them have resulted in development and commercialization of a microbial-based biocontrol product (Charkowski 2018; Czajkowski et al. 2011). Likewise, there are only few examples of the use of such bioproducts being successful in other bacterial pathogen-crop systems (Azizbekyan 2019; Nega 2014; Sheppard et al. 2003) The main reason for this is that for commercialization, the microorganisms need to be effectively formulated in order to remain viable and retain their properties throughout the storage period, in transport and upon application under environmental settings (Berninger et al. 2018).

This study was conducted to develop formulations providing good shelf life of an artificial bacterial consortium termed “The Great Five” (GF) and to test the obtained formulated bacteria for protection of potato tubers against soft rot caused by SRP following long-term (6 months) storage at 8 °C, therefore, mimicking storage conditions present in the commercial potato storage facilities. The biocontrol efficacy of the GF consortium was already reported in our former study, however, only for freshly grown cells and only under short-term storage under disease-favoring conditions (Krzyzanowska et al. 2019a, b. The development of formulations and biocontrol efficacy assays described herein was an important step on the way to prepare “The Great Five” as a commercial product for agricultural applications, as suggested by others (Köhl et al. 2011).

To be eligible for practical application, formulations need to be prepared in a way which allows them to be handled via the standard distribution channels and/or under standard storage conditions (Leggett et al. 2011). This most often involves drying of the product and its storage in low humidity (Rhodes 1993). Desiccation of microorganisms can be carried out in several ways, e. g., freeze-drying, vacuum-drying, spray-drying, fluidized bed-drying, or air-drying (Broeckx et al. 2016); however, freeze-drying (lyophilization) is considered a method of choice as it offers good survival rate, it is applicable both on large and small scale, and results in viable cells that can be rehydrated directly prior to use (Powell 1992; Berninger et al. 2018). The majority of microorganisms when freeze-dried without supplementation of lyoprotectants survive the process poorly (Heckly 1961).Viability rates as low as 0.1% have been frequently reported (Miyamoto-Shinohara et al. 2006).

One of the commonly used lyoprotectants, and the one recommended by American Type Culture Collection (ATCC, Virginia, USA), is Reagent 18. However, the use of Reagent 18 has limitations resulting from the fact that it contains bovine serum albumin (BSA). BSA is an expensive additive, significantly increasing the total cost of Reagent 18. Moreover, it is of animal origin which may lead to ethical concerns. In this study, to overcome these limitations, we developed Reagent PS as a more economically sound and ecofriendly alternative for Reagent 18. In an evaluation experiment performed on 15 microbial strains, the overall survival rate of cells freeze-dried in Reagent PS reached ca. 40–60% and was comparable to that obtained for Reagent 18 (Fig. 1, Supplementary Table S1), while only ca. 5–10% of microorganisms survived without a lyoprotectant (control). Survival rate of ca. 50% was previously reported as a measure of a successful lyophilization (Bozoǧlu et al. 1987).

In the course of this study, the biological control strains of the GF consortium were lyophilized in the newly designed lyoprotectant Reagent PS and subsequently formulated, both as individual strains and as the GF consortium, into two powder formulations. Powder formulations have the widest applications in bioproducts as they can be applied directly on the plant material or, in case of wettable powders, suspended in water and applied as a water-based suspension (Boyetchko et al. 1999). For safety reasons, we selected the latter method of formulation and application. This method is also preferred by farmers as dusting may be hazardous for the workers (Knowles 2008). Although the additives applied to the formulated cells in this study did not offer prolonged shelf life of microorganisms compared with the lyophilizates alone, they considerably reduced their electrostatic properties and dusting, therefore increasing the ease and safety of handling.

As reported earlier, powder formulations in general offer better shelf life than liquid preparations—bacterial cells remain viable for longer periods and the survival is higher than in the other forms of formulations. A crucial factor for successful storage is also the storage temperature. Here, we observed a drastic decline (at average 2.7 log_10_ cfu in the first 6 months) in the viability of the studied strains when the formulations were stored at 22 °C. Storing the product at room temperature is an attractive, cost-efficient option, however rarely possible in case of biological plant protection products. Similar decline of viability in formulated bacterial cells at 22 °C was reported for other biological control agents, including *Pseudomonas fluorescens* EPS62e and *B. subtilis* CPA-8 (Cabrefiga et al. 2014; Yánez-Mendizábal et al. 2012).

In contrast, the GF strains formulated as wettable powders and stored at 8 °C survived for a period of 12 months without a significant drop in cell numbers. Most of the bioformulations currently available on the market have a declared shelf life of 1 year, with minimum of 3 months and up to 6 years in case of selected spore-based products (Arora and Mishra 2016; Preininger et al. 2018). Suggested storage conditions include freezing (− 20 °C), cooling (4–10 °C), or room temperature depending on the type of formulation and its content. This implies that the strains of the GF consortium, formulated to wettable powders as described herein, present shelf life acceptable for commercial products.

Since storage conditions can either positively or negatively influence the activity of biocontrol agents, it is of utmost importance to test bacterial formulations under the appropriate conditions mimicking the real life situation (Costa et al. 2002; Qin et al. 2004). The formulations of GF strains were therefore tested for their biocontrol efficacy against SRP on potato tubers stored for 6 months at 8 °C and under 80% relative humidity. These experimental settings simulate standard (commercial) conditions of potato tuber storage (Bradshaw and Ramsay 2009). For this work, tubers were subsequently inoculated with antagonists and SRPs, stored at 8 °C for 6 months, and then transferred from storage to disease-favorable conditions (28 °C and 85–90% relative humidity) to initiate soft rot symptoms. In this setup, formulations containing antagonistic bacteria decreased symptoms caused by SRP by 50%. Freshly grown cells of the GF consortium, used as reference, offered higher efficiency of protection than formulated bacterial cells of comparable inoculum size. This, however, was not unexpected. It is possible that after rehydration, some strains in the formulations failed to multiply because of their physiological condition. In our study, formulations comprising the GF consortium were freshly prepared from lyophilizates of single bacterial strains. The viable cell count in lyophilizates was determined by dilution plating. During inoculation of potato tubers, water-rehydrated bacterial cells went directly to a poor environment (potato surface, potato skin, and lenticels). We presume that freshly grown cells, coming from optimal growth conditions in a rich medium, may behave differently in this situation than the previously dormant, formulated cells. Similar observations have been made in cases of other formulated biological control agents (Berninger et al. 2018).

Literature suggests that application of microbial consortia, either composed in the laboratory or selected as functional units directly from the environment, may provide a good strategy to develop efficient biocontrol agents (Droby et al. 2016; Fukui et al. 1999; Meyer and Roberts 2002). Currently, the major factor limiting smooth introduction of such products on the market are regulations concerning registration of biological plant protection products, especially in the European Union (Frederiks and Wesseler 2019). According to these regulations, in case of multi-strain products, each active component (strain) should be evaluated separately, significantly increasing the cost, time, and effort necessary to go through the registration procedure, in principal, designed for chemical agents. To register a (bio)product, the applying entity needs to provide data on potential toxicity and ecotoxicity of the product. Currently, there is a strong lobby to alleviate the requirements for registration of biopesticides which, alike bacteria present in the GF consortium, are the elements of the natural microbiome of the soil and/or various plants and which are, according to the current knowledge, not harmful to humans. In the light of growing demand for ecological products for sustainable agriculture (Arora et al. 2016), as well as scientific data on the benefits of applying microbial consortia, it is therefore important that the regulations be adapted to this new generation of products and microbiological agents in general.

In conclusion, in this, study we provided evidence that the newly developed formulations of “The Great Five” micro-consortium, when stored at 8 °C, assure good shelf life of at least 1 year. Moreover, the preserved cells retain their antagonistic activity towards SRP on potato tubers. Further studies are required to optimize the process of application of the formulations under storage conditions. Other matters worth addressing also include the potential of the GF consortium to control SRP under field conditions as well as the longevity of the applied micro-consortium in soil to assure protection on growing plants. Finally, elucidation of the molecular mechanism of antagonism could be of value for using the micro-consortium in other pathogen-host combinations.

### Authors’ contribution statement

TM, DMK, JS, and RC: investigation and methodology; TM, DMK, SJ, and RC: writing, reviewing, and editing the manuscript; TM and DMK: data curation and project administration; SJ and RC: resources; TM, DMK, and JS: visualization; RC: project administration. All authors approved the final version of the manuscript.

## Electronic supplementary material

ESM 1 (PDF 417 kb)
