# Supplementary material for: The Great Five—an artificial bacterial consortium with antagonistic activity towards Pectobacterium spp. and Dickeya spp.: formulation, shelf life, and the ability to prevent soft rot of potato in storage
Source: Appl Microbiol Biotechnol. 2020 Mar 26;104(10):4547–61. doi: 10.1007/s00253-020-10550-x (PMC7190590; doi:10.1007/s00253-020-10550-x)
Supplement: Supplementary file 1 — (PDF 417 kb) [file 253_2020_10550_MOESM1_ESM.pdf]

IFB running title: Antagonistic consortium to control soft rot in storage

The Great Five – an artificial bacterial consortium with antagonistic activity towards *Pectobacterium* spp. and *Dickeya* spp.: formulation, shelf life and the ability to prevent soft rot of potato in storage

SUPPLEMENTARY MATERIAL

Tomasz Maciag [1], Dorota M. Krzyzanowska [1], Sylwia Jafra [1], Joanna Siwinska [2] and Robert Czajkowski [3]*

[1] Laboratory of Biological Plant Protection, Intercollegiate Faculty of Biotechnology, University of Gdansk and Medical University of Gdansk, Gdansk, Poland

[2] Laboratory of Plant Protection and Biotechnology, Intercollegiate Faculty of Biotechnology, University of Gdansk and Medical University of Gdansk, Gdansk, Poland

[3] Laboratory of Biologically Active Compounds, Intercollegiate Faculty of Biotechnology, University of Gdansk and Medical University of Gdansk, Gdansk, Poland

∗ Correspondence:

Robert Czajkowski (Robert.Czajkowski@biotech.ug.edu.pl)

Supplementary Table S1. Survival rate of different microorganisms following freeze drying in water (control) and two lyoprotectants: Reagent 18 and Reagent PS.

		**Survival rate (%) [a]**		
**Species**	**Strain**	**H_2O (ctrl)**	**Reagent 18**	**Reagent PS**
Antagonistic bacteria of the GF consortium				
Serratia plymuthica	A294	8	**61**	**112**
Serratia rubidaea	H440	9	**58**	**112**
Serratia rubidaea	H469	2	**115**	**61**
Rahnella aquatilis	H145	10	**65**	**57**
Enterobacter amnigenus	A167	5	**65**	**50**
Probiotic microorganisms supplemented to humans				
Lactobacillus rhamnosus	GG	3	**65**	**111**
Lactobacillus rhamnosus	573	1	**60**	**111**
Lactobacillus brevis	269Y	5	**72**	**100**
Bacillus coagulans	Colinox	4	**45**	**57**
Saccharomyces boulardi (yeast)	ENTEROL 250	0.04	2	1
Plant-associated bacteria				
Pseudomonas protegens	CHA0	1	**55**	**60**
Ochrobactrum quorumnocens	A44	37	**89**	**59**
Pseudomonas donghuensis	P482	0.05	41	38
Other				
Bacillus subtilis	168	5	**84**	**75**
Escherichia coli	DH5α	**76**	16	13

[a] The survival rate is the ratio between the count of viable cells (cfu per g of bacterial fresh weight) after freeze drying and the same value before the procedure. Each value is an average for three technical replicates. Values equal to or exceeding 50% are shown in bold. The order of strains is determined by the survival rate (highest to lowest) following freeze drying in the lyoprotectant Reagent PS. Values exceeding 100% result from the necessity to sample highly turbid bacterial suspensions (error of the method is estimated to be ca. 10%)

Supplementary Table S2. Count of viable cells for 15 different bacterial strains before and after freeze drying in two different lyoprotectants, Reagent 18 and Reagent PS, and in water (control)

		Before freeze drying			After freeze drying		
**Species**	**Strain**	**H_2O (ctrl)**	**Reagent 18**	**Reagent PS**	**H_2O (ctrl)**	**Reagent 18**	**Reagent PS**
Antagonistic bacteria of the GF consortium							
E. amnigenus	A167	11.04 [a]	11.1	11.45	9.76	10.92	11.15
R. aquatilis	H145	11.07	11.19	10.99	10.07	11	10.75
S. plymuthica	A294	10.96	11.3	10.86	9.87	11.09	10.91
S. rubidaea	H440	10.79	10.55	10.29	9.76	11.32	11.34
S. rubidaea	H469	11.08	11.06	11.22	9.39	11.12	11
Probiotic microorganisms supplemented to humans							
B. coagulans	Colinox	10.43	10.54	10.33	8.47	9.87	9.92
L. brevis	269Y	10.06	9.79	9.85	9.77	10.84	10.98
L. rhamnosus	GG	10.44	10.07	9.97	8.9	10.35	10.38
L. rhamnosus	537	9.99	10.28	10.31	8.4	10.3	10.62
S. boulardi (yeast)	ENTEROL	9.17	9.03	9.43	5.8	7.4	7.4
Plant-associated bacteria							
O. quorumnocens	A44	10.7	10.77	10.77	10.28	10.72	10.54
P. donghuensis	P482	9.82	10.22	10.17	6.78	9.89	9.83
P. protegens	CHA0	10.13	10.28	10.25	8.07	10.03	10.09
Other							

B. subtilis	168	10.68	10.52	10.57	8.74	9.71	9.73
E. coli	DH5α	11.07	10.98	10.98	9.98	9.51	9.3

[a] cell count is expressed in $\log_{10}$ cfu g^{-1} of bacterial fresh weight

Supplementary Table S3. The count of viable cells ($\log_{10}$ cfu mL^{-1} or g^{-1}) in different bacterial formulations stored at 8 °C, depending on the strain, sampling time and the tested lot (Lot 1 and Lot 2).

		**Lot 1 (sampling time in months)** [c]							**Lot 2 (sampling time in months)**							**Slope** [e]		
**Strain**	**Formul.**[a]	**0**	**1**	**2**	**3**	**6**	**9**	**12**	**0**	**1**	**2**	**3**	**6**	**9**	**12**	**Lot1**	**Lot2**	**AVG**[F]
***E. amnigenus* A167**	CTRL	10.30 [b]	9.20	8.71	8.98	8.05	- [d]	-	9.96	10.18	9.13	8.63	8.39	7.75	7.80	-0.392	-0.144	-0.268
	LQ	10.31	9.10	9.13	6.88	8.37	-	-	9.92	8.51	8.99	8.06	9.69	8.71	9.18	-0.323	-0.061	-0.192
	LYO	11.01	11.22	10.95	11.06	10.87	10.58	10.09	10.43	9.45	9.86	9.74	8.83	8.17	7.91	-0.078	-0.048	-0.063
	WP-KAO	11.03	10.92	10.83	10.66	11.00	10.33	10.30	10.21	9.95	9.71	9.66	9.21	7.65	8.10	-0.083	-0.071	-0.077
	WP-DE	10.92	10.88	10.49	10.40	10.52	10.07	9.45	10.21	9.85	9.78	9.58	8.88	8.17	7.61	-0.130	-0.106	-0.118
***R. aquatilis* H145**	CTRL	10.31	8.44	7.68	7.78	7.17	-	-	9.88	9.05	8.65	8.40	7.64	7.47	7.47	-0.317	-0.197	-0.257
	LQ	10.16	8.93	8.92	7.36	8.80	-	-	9.83	8.70	8.92	7.85	8.87	8.39	8.26	-0.325	0.001	-0.162
	LYO	10.97	10.69	10.55	10.32	9.77	9.97	9.65	11.53	10.58	11.36	13.13	10.66	10.12	10.47	-0.079	-0.198	-0.139
	WP-KAO	10.74	10.45	10.24	10.26	10.13	9.48	9.50	11.31	11.01	10.99	13.09	10.26	9.37	9.55	-0.057	-0.204	-0.131
	WP-DE	10.77	10.51	10.04	10.06	10.27	9.53	9.12	11.31	11.12	10.78	12.77	9.99	9.56	9.08	-0.106	-0.216	-0.161
***S. plymuthica* A294**	CTRL	10.31	9.21	8.40	8.82	7.61	-	-	9.62	9.35	9.30	8.94	8.79	8.20	7.81	-0.432	-0.183	-0.308
	LQ	10.09	9.45	9.30	7.35	8.31	-	-	9.57	8.95	9.17	8.71	9.49	9.00	8.26	-0.205	-0.074	-0.140
	LYO	10.00	9.84	10.09	9.65	9.44	9.20	9.16	11.02	10.21	10.71	10.52	10.44	10.29	10.13	-0.102	-0.114	-0.108
	WP-KAO	10.20	9.83	9.56	9.46	9.89	8.90	9.05	10.80	10.14	10.21	10.51	10.21	9.87	9.67	-0.100	-0.197	-0.149

	WP-DE	10.02	10.08	9.37	9.39	9.08	8.61	8.53	10.80	11.20	9.99	10.38	10.30	9.64	9.63	-0.116	-0.216	-0.166
***S. rubidaea* H440**	CTRL	10.44	9.31	8.50	8.58	6.92	-	-	9.68	10.01	9.61	8.76	7.66	4.52	7.91	-0.539	-0.310	-0.425
	LQ	10.50	9.16	8.73	9.49	8.01	-	-	9.63	8.80	9.13	8.30	9.16	8.13	8.83	-0.329	-0.051	-0.190
	LYO	11.53	11.60	11.34	11.45	11.09	10.97	11.16	11.91	10.96	11.60	11.71	11.56	11.32	11.20	-0.044	-0.029	-0.037
	WP-KAO	11.32	11.24	11.21	11.20	11.09	10.85	10.97	11.69	11.32	11.33	11.51	11.17	10.69	10.56	-0.034	-0.088	-0.061
	WP-DE	11.15	11.25	10.88	11.19	11.15	10.77	10.72	11.69	11.73	11.21	11.44	10.99	10.39	10.08	-0.038	-0.139	-0.089
***S. rubidaea* H469**	CTRL	10.37	8.99	8.48	8.48	6.97	-	-	9.79	9.78	9.46	9.05	8.40	7.10	7.03	-0.504	-0.262	-0.383
	LQ	10.38	8.92	8.87	7.88	8.62	-	-	9.74	9.38	9.37	8.44	9.34	8.11	8.11	-0.245	-0.124	-0.185
	LYO	11.60	11.40	11.31	11.31	11.71	10.95	10.63	11.72	11.09	11.77	11.70	11.58	11.29	11.16	-0.065	-0.032	-0.049
	WP-KAO	11.10	10.82	10.90	10.92	10.81	10.35	10.39	11.50	11.35	11.24	11.42	10.98	10.33	9.95	-0.058	-0.131	-0.095
	WP-DE	10.98	10.85	10.50	10.53	10.62	10.01	10.11	11.50	11.84	11.34	11.60	11.14	10.36	9.75	-0.071	-0.160	-0.116
**GF consortium**	CTRL	9.89	9.08	8.63	8.86	8.10	-	-	9.83	9.83	7.15	7.05	6.77	6.24	8.34	-0.257	-0.153	-0.205
	LQ	10.34	9.42	8.39	9.11	8.07	-	-	9.79	9.79	7.03	6.87	7.03	6.82	8.26	-0.322	-0.125	-0.224
	LYO	11.43	11.28	11.03	11.27	10.73	10.74	10.55	11.56	11.56	11.45	11.39	11.04	10.53	10.51	-0.070	-0.102	-0.086
	WP-KAO	10.79	10.87	10.70	10.87	10.91	10.37	10.30	11.34	11.34	10.85	11.09	10.02	10.06	9.11	-0.045	-0.181	-0.113
	WP-DE	11.03	10.98	10.58	10.78	10.71	10.15	10.19	11.34	11.34	10.97	11.23	10.52	10.08	9.62	-0.071	-0.149	-0.110

[a] Formulation: CTRL – cells suspended in ¼ Ringer's buffer; LQ – cells suspended in ¼ Ringer's supplemented with LQ formulation mix. Powder formulations: LYO – bacterial lyophilizates; WP-KAO – bacterial lyophilizates with dry formulation mix and kaolinite carrier; WP-DE – bacterial lyophilizates with dry formulation mix and diatomaceous earth.

[b] Cell count in log_{10} cfu mL^{-1} for the liquid formulations (CTRL and LQ) and log_{10} cfu g^{-1} for the powders (LYO, WP-KAO, WP-DE). Each value is a mean of two technical replicates.

[c] Experiments with Lot 1 and 2 were conducted using different batches of cultures or lyophilizates.

[d] ‘-‘ - not sampled due to low count of viable cell at the previous time point.

[e] Slope of the survival curve expressing the average decline in log_{10} cfu per month of storage. Data to calculate the slope included all time points available for a given set (for the majority of samples 7 time points within the period of 12 months).

[f] AVG – average slope for Lots 1 and 2

Supplementary Table S4. The count of viable cells (log_{10} cfu mL^{-1} or g^{-1}) in different bacterial formulations of Lot 1 stored at 22 °C.

		Sampling time (months)					
**Strain**	**Formulation**[a]	**0**	**1**	**2**	**3**	**6**	**Slope** [c]
***E. amnigenus* A167**	CTRL	10.30 [b]	8.69	8.17	8.19	7.12	-0.454
	LQ	10.31	9.09	7.82	6.88	5.96	-0.708
	LYO	11.01	10.72	9.99	9.21	9.09	-0.338
	WP-KAO	11.03	10.44	9.26	8.80	8.30	-0.454
	WP-DE	10.92	10.32	9.15	8.36	8.70	-0.376
***R. aquatilis* H145**	CTRL	10.31	7.57	7.20	6.88	6.50	-0.504
	LQ	10.16	8.46	8.28	8.11	7.08	-0.433
	LYO	10.97	9.23	7.16	6.65	6.26	-0.736
	WP-KAO	10.74	8.95	6.64	6.11	6.03	-0.735
	WP-DE	10.77	9.04	6.26	6.45	6.00	-0.733
***S. plymuthica* A294**	CTRL	10.31	8.20	7.55	7.89	7.65	-0.329
	LQ	10.09	8.98	9.13	7.10	7.26	-0.473
	LYO	10.00	8.83	8.41	8.52	8.41	-0.205
	WP-KAO	10.20	8.97	8.15	8.06	7.46	-0.407
	WP-DE	10.02	8.27	8.22	7.67	7.58	-0.331
***S. rubidaea* H440**	CTRL	10.44	8.52	7.97	8.27	8.03	-0.297
	LQ	10.50	9.17	8.72	8.29	7.19	-0.504
	LYO	11.53	11.17	10.99	10.69	9.71	-0.299
	WP-KAO	11.32	11.19	10.64	10.49	8.86	-0.419
	WP-DE	11.15	10.90	10.09	10.21	8.91	-0.371
***S. rubidaea* H469**	CTRL	10.37	8.51	8.52	8.41	8.11	-0.282
	LQ	10.38	9.25	9.26	8.67	7.60	-0.424
	LYO	11.60	11.71	10.09	9.65	9.80	-0.341
	WP-KAO	11.10	9.82	9.94	10.00	9.44	-0.206
	WP-DE	10.98	10.29	9.70	9.82	9.68	-0.183

**GF consortium**	CTRL	10.35	8.35	8.01	8.44	7.92	-0.290
	LQ	10.34	9.10	8.84	8.37	7.08	-0.499
	LYO	11.43	10.90	9.69	9.73	9.58	-0.295
	WP-KAO	10.79	10.45	9.34	9.66	8.58	-0.357
	WP-DE	11.03	10.26	9.23	9.38	8.37	-0.413

[a] CTRL – cells suspended in ¼ Ringer's buffer; LQ – cells suspended in ¼ Ringer's supplemented with LQ formulation mix. Powder formulations: LYO – bacterial lyophilizates; WP-KAO – bacterial lyophilizates with formulation mix and kaolinite carrier; WP-DE – bacterial lyophilizates with formulation mix and diatomaceous earth

[b] Cell count in $\log_{10}$ cfu mL^{-1} for the liquid formulations (CTRL and LQ) and $\log_{10}$ cfu g^{-1} for the powders (LYO, WP-KAO, WP-DE). Each value is a mean of two technical replicates.

[c] Slope of the survival curve expressing the average decline in $\log_{10}$ cfu per month of storage. Data to calculate the slope included all time points available for the given set (5 time points within the period of 6 months).
